# Increased Neurotropic Threat from *Burkholderia pseudomallei* Strains with a *B. mallei*–like Variation in the *bimA* Motility Gene, Australia

**DOI:** 10.3201/eid2305.151417

**Published:** 2017-05

**Authors:** Jodie L. Morris, Anne Fane, Derek S. Sarovich, Erin P. Price, Catherine M. Rush, Brenda L. Govan, Elizabeth Parker, Mark Mayo, Bart J. Currie, Natkunam Ketheesan

**Affiliations:** James Cook University, Townsville, Queensland, Australia (J.L. Morris, A. Fane, C.M. Rush, B.L. Govan, E. Parker, N. Ketheesan);; Menzies School of Health Research, Darwin, Northern Territory, Australia (D.S. Sarovich, E.P. Price, M. Mayo, B.J. Currie);; Royal Darwin Hospital, Darwin (B.J. Currie)

**Keywords:** melioidosis, Burkholderia pseudomallei, virulence, neurologic, neurotropism, route of infection, actin-based motility, intracellular, bimA, bacteria, bacterial infection

## Abstract

Neurologic melioidosis is a serious, potentially fatal form of *Burkholderia pseudomallei* infection. Recently, we reported that a subset of clinical isolates of *B. pseudomallei* from Australia have heightened virulence and potential for dissemination to the central nervous system. In this study, we demonstrate that this subset has a *B. mallei*–like sequence variation of the actin-based motility gene, *bimA*. Compared with *B. pseudomallei* isolates having typical *bimA* alleles, isolates that contain the *B. mallei*–like variation demonstrate increased persistence in phagocytic cells and increased virulence with rapid systemic dissemination and replication within multiple tissues, including the brain and spinal cord, in an experimental model. These findings highlight the implications of *bimA* variation on disease progression of *B. pseudomallei* infection and have considerable clinical and public health implications with respect to the degree of neurotropic threat posed to human health.

## Abstract 2

These strains have heightened pathogenic potential for rapid dissemination to multiple tissues, including the central nervous system.

*Burkholderia mallei*, the etiologic agent of glanders, is thought to have evolved from a single strain of *B. pseudomallei*, becoming highly specialized for intracellular persistence (*1*). *B. mallei* and *B. pseudomallei* share sequence similarity and are highly pathogenic through the respiratory route, often initiating rapid disease progression resulting in high mortality (*2*). Unlike *B. pseudomallei*, *B. mallei* has a narrower host range and is less capable of extended persistence in the environment. 

Knowledge of the virulence factors responsible for inducing the diverse spectrum of clinical manifestations of *B. pseudomallei* infection remains limited (*3*). Similar to bacteria of other genera, such as *Listeria*, *Rickettsia*, *Mycobacterium,* and *Shigella*, intercellular and intracellular movement of *Burkholderia* are facilitated by actin polymerization at 1 pole of the bacterium (*4*). The putative autotransporter protein *Burkholderia* intracellular motility A (BimA) has been shown to mediate actin-based motility in *B. pseudomallei* and *B. mallei*, promoting bacterial dissemination while shielding the pathogen from immune surveillance and autophagy (*5*). Differences in the structure of the *bimA* gene in *B. mallei* and *B. pseudomallei* (*6*–*8*) suggest that actin assembly might occur through distinct mechanisms in these 2 *Burkholderia* species. *B. mallei*–like *bimA* variants (*bim_Bm_*) have been identified in a subset of *B. pseudomallei* isolates from Australia and 2 *B. pseudomallei* isolates from India (*9*,*10*). This allele has not yet been identified in isolates from Southeast Asia.

Neurologic melioidosis is a serious, potentially fatal form of *B. pseudomallei* infection. Recently, we reported that although *B. pseudomallei* isolates from patients with neurologic melioidosis do not demonstrate selective neurotropism in an experimental model, a distinct subset of *B. pseudomallei* isolates appeared equipped for rapid dissemination to multiple tissues, including the central nervous system (CNS), after infection (*11*). Correlation of virulence genes of *B. pseudomallei* with clinical presentations of melioidosis identified the *bim_Bm_* allele as a risk factor for neurologic melioidosis (*12*). Given the importance of BimA in intercellular and intracellular spread of *Burkholderia* spp. and the recognition of *bim_Bm_* variants of *B. pseudomallei* in northern Australia, we hypothesized that *bim_Bm_* variants of *B. pseudomallei* would have an increased advantage for establishment of infection and dissemination compared with typical *bim_Bp_* strains. Therefore, we used a well-characterized animal model of melioidosis to compare virulence and disease progression after infection with clinical isolates of *B. pseudomallei* collected in the Northern Territory of Australia during October 1989–October 2012 and identified as having either the *bim_Bm_* or *bim_Bp_* allele (*13*).

## Methods

### *B. pseudomallei* Isolates

*B. pseudomallei* strains were isolated from patients with melioidosis. Clinical details and the sequence type determined from multilocus sequence typing of the *B. pseudomallei* strains investigated are noted (Table). Additional details are described elsewhere (*11*,*12*,*14*,*15*). These isolates were chosen to represent *B. pseudomallei* strains previously identified as having *bim_Bm_* (n = 7) and *bim_Bp_* (n = 8) alleles within the *bimA* gene (*10*,*12*).

### Animal Infection

We used 8- to 12-week-old C57BL/6 and BALB/c mice purchased from the Small Animal Breeding Facility at James Cook University. Experiments were approved by the Institutional Animal Ethics committee (A1500). To mimic natural routes of infection, intranasal or subcutaneous routes were used for inoculation by using methods described previously (*16*). *B. pseudomallei* isolates were cultured to logarithmic phase and prepared for inoculations as previously described (*11*).

### Virulence Determination

Virulence of *bim_Bm_* (n = 7) and *bim_Bp_* (n = 6) isolates were compared in mice as described previously (*11*). The 50% infectious dose (ID_50_) was determined by using a modified version of the Reed and Meunch method (*17*). Virulence, as defined by the ID_50_ values for *B. pseudomallei* strains, were compared in BALB/c and C57BL/6 mice after intranasal and subcutaneous infection. Data for *bim_Bm_* and *bim_Bp_* strains are expressed as mean log_10_ ID_50_ +SD

### Bacterial Dissemination and Disease Progression

We selected *bim_Bm_* (MSHR543) and *bim_Bp_* (MSHR305) strains of comparable virulence (determined by intranasal ID_50_ values as 2.6 × 10^2^ CFU and 2.9 × 10^2^ CFU, respectively) for comparison of bacterial dissemination after intranasal infection of C57BL/6 mice. C57BL/6 mice provide a more accurate model for neurologic melioidosis because this form of the disease tends to occur in otherwise healthy persons without known risk factors (*13*). MSHR543 (*bim_Bm_*) was isolated from a localized skin infection in a healthy 22-year-old with a cut on her hand that was exposed to muddy water. Blood cultures were negative, and she remained systemically well with no evidence of dissemination of *B. pseudomallei.* The *bim_Bp_* (MSHR305) strain was isolated from a patient with a fatal case of neurologic melioidosis. The 64-year-old patient had a history of excessive alcohol consumption and had had onset of flaccid paralysis after a period of influenza-like illness (*14*). An equivalent dose of MSHR543 (1.4 × 10^4^ CFU) or MSHR305 (1.1 × 10^4^ CFU) was used to inoculate mice. Survival rates and signs of disease were monitored daily for a period of 21 days (n = 10 mice per isolate). Mice that became moribund during the experimental period were euthanized, and bacterial loads were determined in organs and pathology of CNS investigated. Parallel groups of mice were inoculated with MSHR543 (*bim_Bm_*) (n = 15) and MSHR305 (*bim_Bp_*) (n = 15) for assessment of bacterial loads within blood, liver, spleen, lung, cervical lymph node , brain, and nasal-associated lymphoid tissue (NALT) at 2 hours, 1 day, and 3 days postinfection (n = 5 mice per time point) by using methods described previously (*11*). The detection limit of bacteria in blood and organs was 2 CFU. Data are expressed as the mean log_10_ CFU +SD.

### Bacterial Growth Rate

The growth of *B. pseudomallei* isolates in trypticase soy broth (TSB) was compared. Overnight broth cultures of *B. pseudomallei* isolates were diluted 1:10 in fresh TSB and incubated in triplicate at 37°C with shaking at 120 rpm. Absorbance (600 nm) was measured hourly for 10 hours with a microplate reader (Fluostar Omega; BMG Labtech, Mornington, VIC, Australia) and the exponential growth rate for each isolate determined. Data are presented as the mean gradient (μhr^–1^) +SD for *bim_Bm_* and *bim_Bp_* strains.

### Internalization and Persistence of Bacteria in Phagocytic Cells

We determined internalization and intracellular persistence of *B. pseudomallei* isolates (n = 7 *bim_Bm_*; n = 8 *bim_Bp_*) in mononuclear phagocytes after co-culture with murine leukocytes. Leukocytes were isolated from spleen and peripheral lymph nodes (cervical, mediastinal, axillary, inguinal, and popliteal) of uninfected female C57BL/6 mice (*18*). *B. pseudomallei* isolates were grown to logarithmic phase, washed then added to leukocyte cultures at a multiplicity of infection of 1 (mononuclear cell): 5 (bacteria) (*19*). After 2 hours of co-culture, kanamycin (250 μg/mL) was added to wells to limit extracellular bacterial growth (*18*). Internalization (2 h) and persistence (8 and 24 h) of *B. pseudomallei* isolates in leukocytes was determined by flow cytometry. Uninfected and *B. pseudomallei*–infected leukocytes were fluorescently stained with a combination of anti-mouse fluorescein isothiocyanate–conjugated CD45 and F4/80 (BD Biosciences, North Ryde, NSW, Australia) and peridinin chlorophyll-cyanine 5.5 (PerCP-Cy5.5)–conjugated CD11c (eBioscience, San Diego, CA, USA) by using methods described previously (*18*). After fixation and permeabilization, leukocytes were stained with polyclonal rabbit anti–*B. pseudomallei* outer membrane protein antibody (BpOMP). A secondary biotinylated goat anti-rabbit IgG (Vector Labs, Burlingame, CA, USA) monoclonal antibody and streptavidin–phycoerythrin conjugate (eBioscience) was used for detection of the primary antibody. Acquisition (2 × 10^5^ leukocytes) was performed by using a FACSCalibur with Cell Quest software (BD Biosciences) and FlowJo software (Tree Star, Inc., San Carlos, CA, USA) was used for postacquisition analysis. The fluorescence of extracellular bacteria was quenched with Trypan blue (0.2%). Data are expressed as the percentage or total number of leukocytes (CD45^+^), macrophages (F4/80^+^), or dendritic cells (CD11c^+^) positive for intracellular BpOMP staining. Two independent experiments were conducted, and the mean +SD of data from both experiments is shown. Microbiologic culture was used to confirm intracellular *B. pseudomallei* numbers estimated by BpOMP staining (*20*).

### Statistical Analysis

We performed statistical analysis by using Graphpad Prism Version 6 (Graphpad Software, La Jolla, CA, USA) and used Kaplan–Meier survival curves to compare susceptibility to infection with *B. pseudomallei* isolates. Virulence parameters (ID_50_ values, time for development of neurologic symptoms, and intracellular bacterial loads within leukocytes) for *bim_Bm_* and *bim_Bp_* strains were compared by using the Mann-Whitney U test. Bacterial load kinetics in organs after infection with MSHR543 (*bim_Bm_*) and MSHR305 (*bim_Bp_*) were tested for significance using 2-way analysis of variance with Sidak’s post hoc analysis. We considered comparisons significant at p<0.05.

## Results

### High Virulence of *Bim_Bm_* Variants in Murine Models of Melioidosis

**Figure 1:**
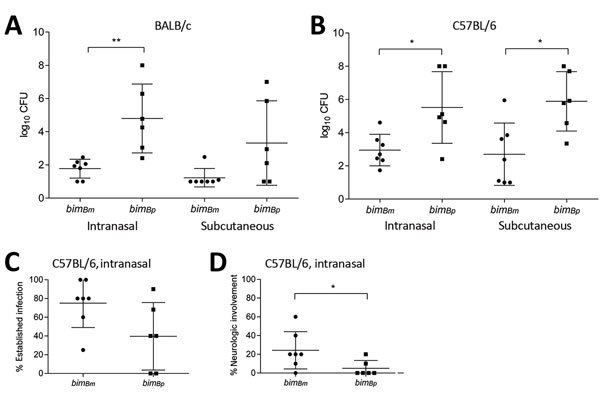
Virulence of *bim_Bm_* and *bim_Bp_ Burkholderia pseudomallei* isolates. Day 21 50% infectious dose values after intranasal and subcutaneous infection of BALB/c (A) and C57BL/6 (B) mice with *bim_Bm_* (n = 7) and *bim_Bp_* (n = 6) *B. pseudomallei* isolates. Groups of 5 mice were inoculated via intranasal and subcutaneous routes at 10-fold increasing doses of *B. pseudomallei*, ranging from 10^0^ CFU to 10^7^ CFU. Virulence of *bim_Bm_* isolates was significantly greater for both mouse strains, regardless of the infection route. Data are expressed as mean log_10_ CFU +SD. C57BL/6 mice (n = 10) were infected with *bim_Bm_* (n = 7) and *bim_Bp_* (n = 6) *B. pseudomallei* isolates at equivalent doses (10^4^ CFU) and monitored for 21 days postinfection. The percentage of mice for a given bacterial strain for which evidence indicated establishment of *B. pseudomallei* infection (culture-positive growth from tissues) (C) and signs of neurologic involvement (e.g., head tilt, spinning behavior, and hind leg paresis) (D) was increased for animals exposed to *bim_Bm_* compared with *bim_Bp_* isolates. *p<0.05; **p<0.01.

We compared virulence, as defined by ID_50_, for *bim_Bm_* and *bim_Bp_* strains in *B. pseudomallei*–susceptible (BALB/c) and *B. pseudomallei*–partially resistant (C57BL/6) mice after intranasal and subcutaneous infection (*16*,*21*). *B. pseudomallei bim_Bm_* strains were significantly more virulent for BALB/c and C57BL/6 (Figure 1, panels A and B) mice than *bim_Bp_* strains, regardless of route of infection. These findings are consistent with the BALB/c–C57BL/6 model of contrasting resistance to *B. pseudomallei* (*21*).

When equivalent inoculating doses of *B. pseudomallei* strains were compared (10^4^ CFU), *bim_Bm_* strains were more likely to establish persistent infection with bacteria recoverable from multiple organs at 21 days postinfection after intranasal infection of C57BL/6 mice (p = 0.077) (Figure 1, panel C). Additionally, neurologic involvement occurred with more frequency in animals infected through the intranasal route with *bim_Bm_* compared with those infected with *bim_Bp_* strains when an equivalent inoculating dose (10^4^ CFU; n = 10 mice/*B. pseudomallei* strain) was used (p = 0.046) (Figure 1, panel D). Most *B. pseudomallei* strains tested were capable of CNS infection; however, neurologic involvement tended to occur at comparatively lower inoculating doses for *bim_Bm_* than *bim_Bp_* strains. The mean number of bacteria required to infect C57BL/6 mice through the respiratory tract and result in the development of neurologic signs in >20% of mice was 9 × 10^3^ CFU (range 5.3 × 10^1^ to 2 × 10^4^ CFU) for *bim_Bm_* and 3.7 × 10^5^ CFU (range 2.6 × 10^4^ to 6.6 × 10^5^) for *bim_Bp_* (p = 0.048). Despite infection of C57BL/6 mice with doses as high as 10^8^ CFU, neurologic symptoms were never observed after infection with 2 strains (MSHR3709 and MSHR1655), both of which are type *bim_Bp_*.

The mean number of bacteria required to infect susceptible BALB/c mice via the respiratory route and manifest neurologic signs in >20% of mice was 8.6 × 10^3^ CFU (range 4 × 10^1^ to 3 × 10^4^ CFU) for *bim_Bm_* and 1.5 × 10^5^ CFU (range 2.6 × 10^4^ to 4.2 × 10^5^ CFU) for *bim_Bp_* (p = 0.03). For C57BL/6 mice, the mean number of days postinfection for onset of neurologic symptoms was 9 (range 5–16) days; for BALB/c mice, it was 11 (range 4–18) days. These findings indicate that *bim_Bm_* variants are significantly more virulent than *bim_Bp_* strains in murine models of melioidosis and suggest that fewer inoculating bacteria are required to establish CNS infection.

### Differing Disease Progression for *bim_Bm_* and *bim_Bp_* Strains after Intranasal Infection

**Figure 2:**
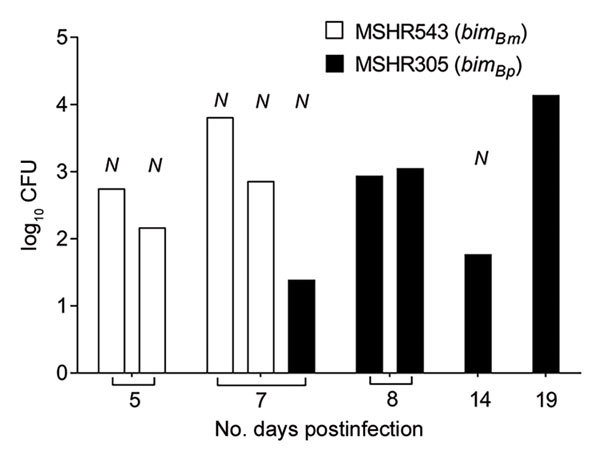
Brain bacterial loads in mice that had signs of neurologic involvement and succumbed to infection with MSHR543 (*bim_Bm_*) and MSHR305 (*bim_Bp_*) *Burkholderia pseudomallei* isolates. Bacterial loads in brains of C57BL/6 mice (MSHR543, n = 4; MSHR305, n = 5) that had become moribund and required euthanasia within the 21-day experimental period after intranasal infection with MSHR543 (1.4 × 10^4^ CFU; white bars) and MSHR305 (1.1 × 10^4^ CFU; black bars). *N* indicates mice that displayed symptoms of neurologic involvement. Data are expressed as log_10_ CFU. Mice exposed to MSHR543 (*bim_Bm_*) had signs of neurologic involvement and became moribund within 7 days of exposure.

**Figure 3:**
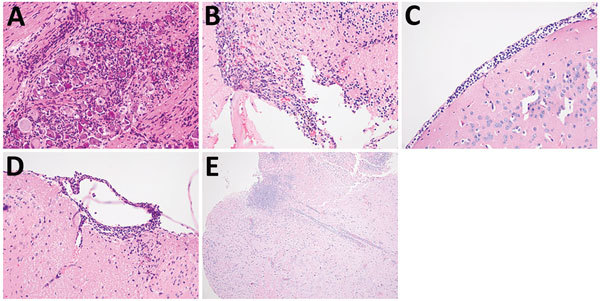
Central nervous system pathology in mice that had signs of neurologic involvement and succumbed to infection with *bim_Bm_* and *bim_Bp_ Burkholderia pseudomallei* isolates. Evidence of central nervous system pathology was demonstrated in these mice. Inflammatory infiltrates were prominent in trigeminal nerve branches and ganglion (original magnification ×400) (A) and in the olfactory bulb (original magnification ×200) (B). Cranial meningitis (C) and spinal (D) meningitis were observed, often with involvement of underlying parenchyma (original magnification ×400). Microabscesses were frequently observed in cerebral cortex (original magnification ×100) (E), brainstem (not shown) and cerebellum (not shown) of mice that had neurologic symptoms and succumbed to infection.

We selected a *bim_Bm_* (MSHR543) and *bim_Bp_* (MSHR305) strain of comparable virulence to compare organ tropism after intranasal infection (intranasal ID_50_ values of 2.6 × 10^2^ and 2.9 × 10^2^ CFU, respectively). Twenty-one day mortality rates were comparable after intranasal infection with either MSHR543 (*bim_Bm_*) or MSHR305 (40% and 50%, respectively). However, of the animals monitored for survival, 2 of the 5 mice that succumbed to infection with MSHR305 (*bim_Bp_*) had neurologic symptoms (1 with head tilt on day 7, another with hind limb paresis on day 14). In contrast, all of the 4 mice that succumbed to infection with MSHR543 (*bim_Bm_*) had symptoms of neurologic melioidosis (3 with head tilt on day 5 and day 7, the other with hind leg paresis on day 7). Moribund mice were euthanized and tissues processed for bacterial load determination. Bacterial loads were high in brains of moribund mice (Figure 2). *B. pseudomallei* was typically recovered from all tissues investigated, although levels tended to be low or undetectable in the blood of moribund mice that had signs of neurologic infection in the first week postinfection. Compared with moribund animals infected with MSHR543 (*bim_Bm_*), bacterial loads were significantly higher in NALT of moribund mice infected with MSHR305 (*bim_Bp_*, p = 0.025), with a similar trend observed in lung. Abscessation was observed in the nasal epithelium, with extensive suppurative inflammation in the olfactory submucosa extending to the olfactory bulb and moderate infiltration in the trigeminal nerve branches (Figure 3, panels A and B) in mice that had signs of neurologic involvement at day 5 postinfection with MSHR543 (*bim_Bm_*). Leptomeningitis and encephalomyelitis were cardinal features in these animals (Figure 3, panels C and D). We also observed cranial microabscesses were in animals that succumbed to infection, although the area affected varied and included the cerebellum, brainstem, and cerebral cortex (Figure 3, panel E).

**Figure 4:**
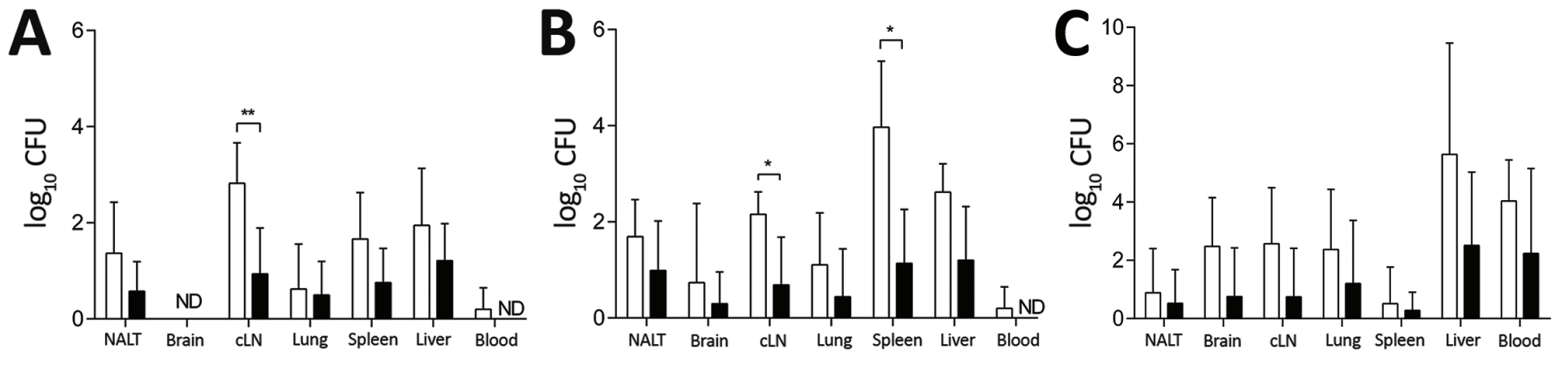
Comparison of early bacterial dissemination and persistence after intranasal infection of C57BL/6 mice with MSHR543 (*bim_Bm_*) and MSHR305 (*bim_Bp_*) *Burkholderia pseudomallei* isolates. A, B) Bacterial load at day 1 (A) and day 3 (B) postinfection in nasal-associated lymphoid tissue, brain, cervical lymph nodes, lung, spleen, liver, and blood after intranasal infection of C57BL/6 mice (n = 5/time point) with MSHR543 (1.4 × 10^4^ CFU; white bars) and MSHR305 (1.1 × 10^4^ CFU; black bars). C) Bacterial organ loads in mice that survived the 21-day experimental period (MSHR543, n = 6; MSHR305, n = 5). Data are expressed as mean log_10_ CFU + SD (upper bars only). cLN, cervical lymph nodes; NALT, nasal-associated lymphoid tissue; ND, not detected. *p<0.05; **p<0.01.

Systemic dissemination occurred rapidly for MSHR543 (*bim_Bm_*) and MSHR305 (*bim_Bp_*); bacteria were recovered from multiple sites by day 1 postinfection (Figure 4). At 2 hours postinfection, NALT was the only tissue that bacteria were cultured from, with levels comparable for mice infected with MSHR543 (*bim_Bm_*) and MSHR305 (*bim_Bp_*) (log_10_ CFU of 0.9 +1.1 and 0.3 +1.1, respectively). Compared with MSHR305 (*bim_Bp_*), replication of MSHR543 (*bim_Bm_*) was significantly higher in cervical lymph nodes and spleen (Figure 4). Bacterial loads were low in brains of mice infected with MSHR543 (*bim_Bm_*) and MSHR305 (*bim_Bp_*) within 3 days of infection despite signs of neurologic involvement by day 5 postinfection in 4 mice infected with MSHR543 (*bim_Bm_*), corresponding to bacterial loads in the brain in excess of 10^2^ CFU (Figure 2). In comparison, only 1 animal infected with MSHR305 (*bim_Bp_*) had symptoms of neurologic melioidosis and required euthanasia within 7 days.

Five mice (50%) survived to 21 days after intranasal infection with MSHR305 (*bim_Bp_*), of which 4 had evidence of persistent *B. pseudomallei* infection, with bacteria recovered from the brain of 1 mouse (Figure 4, panel C). Six mice (60%) survived after intranasal infection with MSHR543 (*bim_Bm_*), and all had evidence of persistent infection, with bacteria recovered from the brains of 5 mice (Figure 4, panel C).

These findings demonstrate that despite equivalent inoculating doses and similar 21-day mortality rates, the pattern and kinetics of dissemination differ for MSHR543 (*bim_Bm_*) and MSHR305 (*bim_Bp_*) after intranasal infection, with neurologic involvement occurring with more frequency after infection with MSHR543 (*bim_Bm_*).

### Increased Persistence of *bim_Bm_* Strains in Mononuclear Phagocytic Cells

**Figure 5:**
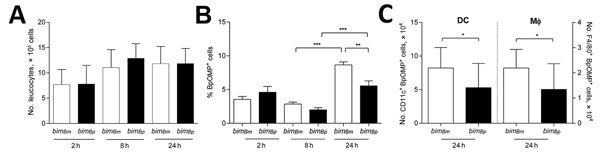
Internalization and persistence of *bim_Bm_* and *bim_Bp_ Burkholderia pseudomallei* isolates within murine leukocytes. Spleen and lymph node–derived leukocytes were co-cultured with *B. pseudomallei* isolates at multiplicity of infection 1:5. A) At 2, 8, and 24 hours postinfection, absolute numbers of CD45^+^ leukocytes were comparable in cultures infected with *bim_Bm_* and *bim_Bp_* strains. B) Bacterial uptake (2 h) and persistence (8 h and 24 h) was compared by assessing the percentage of CD45^+^ leukocytes that were positive for intracellular *B. pseudomallei* outer membrane protein antibody (BpOMP) staining using flow cytometry. BpOMP staining increased within leukocytes between 8 hours and 24 hours of cultures. Compared with *bim_Bp_*, the percentage of leukocytes positive for intracellular BpOMP was significantly higher in cultures stimulated with *bim_Bm_* isolates at 24 hours postinfection. Internalization of *bim_Bm_* or *bim_Bp_* isolates by CD11c^+^ dendritic cells and F4/80^+^ macrophages was comparable (not shown). C) However, persistence of *bim_Bm_* strains was significantly higher in dendritic cells and macrophages after 24 hours of culture. Data reflect the mean +SD of 2 independent experiments. BpOMP, *B. pseudomallei* outer membrane protein antibody; DC, dendritic cells; Mϕ, macrophages. *p<0.05; **p<0.01; ***p<0.001.

To investigate whether differences observe in systemic dissemination in vivo might be attributable to inherent differences in multiplication of *bim_Bm_* and *bim_Bp_* strains, we compared the in vitro growth rate of isolates in broth culture. No significant differences were observed for the exponential growth of *bim_Bm_* and *bim_Bp_* variants in TSB (slope, μhr^-1^, 0.105 +0.02 and 0.092 +0.02, respectively). Having demonstrated that *bim_Bm_* and *bim_Bp_* strains multiply at the same rate in cell-free media, we next investigated whether intracellular growth rates were comparable for the 2 groups of isolates. Because macrophages and dendritic cells play a pivotal role in protection against *B. pseudomallei* infection (*3*), we compared the uptake and persistence of *bim_Bm_* (n = 7) and *bim_Bp_* (n = 8) isolates in ex vivo cultures of murine spleen and lymph node–derived macrophages and DC. Absolute numbers of leukocytes were comparable for *bim_Bm_*- and *bim_Bp_*-infected cultures at 2, 8, and 24 hours postinfection (Figure 5, panel A). The percentage of leukocytes positive for BpOMP staining was also comparable in cultures infected with *bim_Bm_* and *bim_Bp_* strains at 2 and 8 hours postinfection (Figure 5, panel B). However, by 24 hours, the proportion of BpOMP^+^ leukocytes was significantly higher in cultures infected with *bim_Bm_* than *bim_Bp_* strains (p = 0.002), and persistence of *bim_Bm_* isolates was greater in CD11c^+^ dendritic cells (p = 0.012) and F4/80^+^ macrophages (p = 0.006) than *bim_Bp_* strains (Figure 5, panel C). Overall, these data suggest that *bim_Bm_* strains of *B. pseudomallei* might possess mechanisms to facilitate their internalization and intracellular persistence within professional phagocytes.

## Discussion

Although uncommon, neurologic melioidosis is a severe and debilitating form of *B. pseudomallei* infection, primarily affecting healthy persons with no recognizable risk factors and occurring with increased frequency in Australia (*13*,*14*,*22*). Diagnosis and management of neurologic melioidosis is challenging because of nonspecific clinical presentation, poor diagnostics, and intrinsic resistance to antibiotics. Similar to other intracellular bacteria, *B. pseudomallei* and *B. mallei* are able to spread to adjacent host cells and evade immune surveillance through the formation of actin tails in a process that involves polymerization of host actin monomers (*5*,*10*,*23*,*24*). Polymorphisms in machinery used for actin assembly in other obligate intracellular bacteria have been reported to influence virulence and tissue tropism (*25*–*27*). Recently, isolates possessing a *B. mallei*–like *bimA* allele (*bim_Bm_*) were shown to be associated with neurologic involvement in human melioidosis (*12*). Our study provides in vivo evidence of the implications of the *bim_Bm_* sequence variation on disease progression and severity of experimental melioidosis. Compared with *B. pseudomallei* isolates with typical BimA motifs, *bim_Bm_* variants were more virulent in an animal model of melioidosis when delivered intranasally or subcutaneously. This subset of strains was associated with increased persistence within phagocytic cells and increased likelihood of establishing CNS infection compared with *bim_Bp_* strains of *B. pseudomallei*.

Although no evidence from our study indicates preferential seeding of the CNS compared with other tissues, CNS infection did occur with increased frequency and at lower inoculating doses after infection of mice with *bim_Bm_* than *bim_Bp_* strains of *B. pseudomallei*. Neurologic involvement was observed after intranasal and subcutaneous inoculation with *B. pseudomallei* isolates, although the frequency of CNS infection increased after intranasal infection. Neurologic involvement, as evidenced by bacterial colonization of the brain and neutrophil infiltration to the cranial and spinal meninges, occurred with more frequency in animals infected with MSHR543 (*bim_Bm_*) than those exposed to MSHR305 (*bim_Bp_*). Although we observed considerable variability in the sites of abscessation in the CNS, leptomeningitis, meningoencephalitis, and encephalomyelitis were common features in animals that succumbed to infection. Similar neuropathology has been reported in experimental models using intravenous (rather than intranasal) challenge of mice with *B. pseudomallei* (*28*). Furthermore, the neuropathology observed in our study is consistent with the only published histopathologic study of human CNS from patients with melioidosis encephalomyelitis (*14*).

Clinical and experimental data suggest *B. pseudomallei* is capable of using >1 mechanism for entry into the brain and spinal cord (*28*–*37*). *B. pseudomallei* has been shown to take advantage of olfactory and trigeminal nerve branches to gain direct access to the brain after respiratory infection of mice (*29*–*32*), and St. John et al. (*32*) recently demonstrated a role for *bimA* in direct CNS invasion by *B. pseudomallei*. Clinical reports also support progression of sinusitis or upper respiratory tract infection with *B. pseudomallei* to neurologic melioidosis (*33*–*35*). Additionally, cortical brain abscesses, a clinical presentation commonly reported for neurologic melioidosis in Southeast Asia (*33*), were observed and are consistent with bacteremic spread of *B. pseudomallei*, directly or through transmigration of infected leukocytes, to the CNS (*28*). In addition to direct infection through the upper respiratory tract, cases of neurologic melioidosis from the Darwin Prospective Melioidosis Study have recently provided strong support for direct brainstem or spinal cord infection occurring through nerve root translocation of bacteria secondary to skin inoculation with *B. pseudomallei* on the face/scalp or limbs (*36*,*37*). The observation of hind leg paraparesis in some animals after *B. pseudomallei* infection in our study provides additional support for this postulated mechanism of CNS entry.

In our study, rapid systemic dissemination to secondary lymphoid tissues was observed for *B. pseudomallei* *bim_Bm_* and *bim_Bp_* variants, with significantly higher bacterial loads observed earlier in these tissues after infection with the *bim_Bm_* variant. Moreover, despite significant reduction in intracellular bacterial loads, persistence of *B. pseudomallei* was evident in vitro in dendritic cells and macrophages, tissue phagocytic cells that *B. pseudomallei* would be exposed to in the early stages of subcutaneous and intranasal infection. We acknowledge that other leukocyte subsets might support intracellular infection with *B. pseudomallei* and therefore potentially contribute to rapid dissemination of this bacterium in vivo. We limited our assessment to dendritic cells and macrophages because these cells are among the earliest responders to infection and are critical for controlling *B. pseudomallei* infection (*3*,*20*,*21*). Skin dendritic cells also migrate to secondary lymphoid tissues, facilitating the trafficking and systemic dissemination of live intracellular *B. pseudomallei* (*18*). Our data support a potential role for professional phagocytic cells in rapid systemic dissemination of *B. pseudomallei* to distant sites such as the CNS. 

As an increasing number of clinically derived strains are genotyped, it is becoming apparent that the manifestations of melioidosis are likely to be influenced by the infecting strain, as well as the route of infection, infecting dose, and host risk factors for melioidosis. Our findings from this current study provide strong support to our clinical observations (*12*) that *bim_Bm_* variation is a predictor for severe forms of melioidosis, including neurologic involvement. Despite comparative interrogation of genomes between *B. pseudomallei* strains of contrasting virulence (*38*,*39*), to date *bimA* has been identified as the only gene with a strong association with neurologic melioidosis. However, our observation that *bim_Bp_* strains have the potential to invade the CNS, albeit typically at higher inoculating doses than *bim_Bm_* strains, suggest that genes other than *bimA* also contribute to *B. pseudomallei* invasion and dissemination in vivo*.* Under favorable circumstances, avirulent *B. pseudomallei* strains and even the closely related but avirulent bacterium, *B. thailandensis*, can initiate systemic and lethal infection (*40*,*41*). Identifying and characterizing bacterial effector proteins involved in the intracellular and intercellular spread and persistence of *B. pseudomallei* and *B. mallei* will be critical for identification of novel agents to manipulate these processes with therapeutic application.

**Table:** Clinical and patient characteristics and sequence type diversity of *bim_Bm_* and *bim_Bp_ Burkholderia pseudomallei* isolates, Australia

Isolate no.	Age, y/sex	Risk factors	Clinical presentation	Outcome	MLST genotype
*bim_Bm_*					
MSHR62	23/M	None	Brainstem encephalitis	Survived	148
MSHR435	37/M	None	Brainstem encephalitis	Survived	126
MSHR543	22/F	None	Skin ulcer	Survived	294
MSHR668	53/M	None	Diffuse encephalitis	Survived	129
MSHR1153	59/M	DBT	Brainstem encephalitis	Died	117
MSHR2138	49/F	DBT	Bacteremia	Survived	456
NCTC13178	6/M	None	Brainstem encephalitis	Died	286
bim_Bp_					
MSHR305	64/M	ALC	Encephalitis, myelitis	Died	36
MSHR346	49/M	ALC, COPD	Pneumonia	Survived	243
MSHR465	67/M	DBT, COPD	Pneumonia, septic shock	Died	132
MSHR1655	61/F	COPD	Pneumonia	Survived	131
MSHR3709	14/M	None	Brainstem encephalitis	Survived	132
MSHR974*	16/F	None	Skin ulcer	Survived	554
MSHR4237*	45/F	None	Pneumonia	Survived	868
NCTC13179	54/M	DBT	Skin ulcer	Survived	613

*Additional isolates included for internalization and persistence assays. ALC, hazardous alcohol use; COPD, chronic obstructive pulmonary disease; DBT, diabetes; MLST, multilocus sequence typing.
